# COMPREHENSIVE MALNUTRITION ASSESSMENT IMPROVES HEALTH OUTCOMES AMONG HOMEBOUND OLDER ADULTS

**DOI:** 10.1093/geroni/igad104.3243

**Published:** 2023-12-21

**Authors:** Susan Saffel-Shrier, Amy Covington, jing Wang

**Affiliations:** University of Utah, Salt Lake City, Utah, United States; University of Utah, Salt Lake City, Utah, United States; University of Utah, Salt Lake City, Utah, United States

## Abstract

Approximately 30 percent of older adults are malnourished upon hospital admission while others develop malnutrition during hospitalization and subsequent discharge. High hospital readmission rates are associated with malnutrition. Malnutrition is common among older adults discharged to home and receiving home delivered meals. However, there is limited research on the appropriate malnutrition care for this population. The aim of this study was to investigate the effectiveness of a novel social determinants of health (SDOH)-based comprehensive malnutrition assessment (CMA) on malnutrition treatment and functionality. A pilot study of 46 participants recruited from northern Utah Area Agencies on Aging Nutrition Programs was conducted. The SDOH-CMA was performed during home visitations by a registered dietitian nutritionists for both the intervention and control groups over a six-month period. Logistic regression was then conducted to compare the effect of the SDOH-based CMA on malnutrition as assessed by the Nutrition-Focused Physical Exam (NFPE) between intervention and control groups. Odds ratios (OR) with p values were reported. At baseline, 91.4% of participants were white, 54% were female, 76% were unmarried, 67% lived below the federal poverty level, 61% lived alone, 51% received home health, 51% had anxiety, 33% had PHQ-2 screened depression, 22% were unable to cook, and 13% were unable to shop. The intervention group had significant improvements in the NFPE (OR=10.1, p=.02), calorie intake (OR= 19.6, p=.02), protein intake (OR=36.7, p =.007) and the Timed Up and Go test (OR=7.7, p=.05) than the control group. The intervention was significantly effective in improving nutritional status and functionality.

